# Spatial heterogeneity distribution of soil total nitrogen and total phosphorus in the Yaoxiang watershed in a hilly area of northern China based on geographic information system and geostatistics

**DOI:** 10.1002/ece3.2410

**Published:** 2016-09-01

**Authors:** Yu Liu, Peng Gao, Liyong Zhang, Xiang Niu, Bing Wang

**Affiliations:** ^1^ Forestry College Mountain Tai Forest Ecosystem Research Station of State Forestry Administration Shandong Provincial Key Laboratory of Soil Erosion and Ecological Restoration Shandong Agricultural University Tai'an Shandong China; ^2^ Research Institute of Forest Ecology, Environment and Protection Chinese Academy of Forestry Beijing Collaborative Innovation Center for Eco‐Environmental Improvement with Forestry and Fruit Trees Beijing China

**Keywords:** geographic information system, geostatistics, soil total nitrogen, soil total phosphorus, spatial heterogeneity distribution, Yaoxiang watershed

## Abstract

Soil total nitrogen (STN) and total phosphorus (STP) are important indicators of soil nutrients and the important indexes of soil fertility and soil quality evaluation. Using geographic information system (GIS) and geostatistics, the spatial heterogeneity distribution of STN and STP in the Yaoxiang watershed in a hilly area of northern China was studied. The results showed that: (1) The STN and STP contents showed a declining trend with the increase in soil depth; the variation coefficients (*C*_v_) of STN and STP in the 0‐ to 10‐cm soil layer (42.25% and 14.77%, respectively) were higher than in the 10‐ to 30‐cm soil layer (28.77% and 11.60%, respectively). Moreover, the *C*_v_ of STN was higher than that of STP. (2) The maximum *C*_0_/(*C*_0_ + *C*_1_) of STN and STP in the soil layers was less than 25%, this indicated that a strong spatial distribution autocorrelation existed for STN and STP; and the STP showed higher intensity and more stable variation than the STN. (3) From the correlation analysis, we concluded that the topographic indexes such as elevation and slope direction all influenced the spatial distribution of STN and STP (correlation coefficients were 0.688 and 0.518, respectively). (4) The overall distribution of STN and STP in the Yaoxiang watershed decreased from the northwest to the southeast. This variation trend was similar to the watershed DEM trend and was significantly influenced by vegetation and topographic factors. These results revealed the spatial heterogeneity distribution of STN and STP, and addressed the influences of forest vegetation coverage, elevation, and other topographic factors on the spatial distribution of STN and STP at the watershed scale.

## 1 Introduction

As the basic building blocks of all known forms of life on Earth, soil total nitrogen (STN) and total phosphorus (STP) are significant evaluation indexes of soil fertility in the terrestrial ecosystem (Gao, Yang, & Liu, 2015; Zhang, Cha, & Shen, 2011). Fully understanding the characteristics of their spatial heterogeneity distribution at the watershed scale is the basis of reasonable evaluation to soil nutrients. In addition to providing nutrient sources for vegetation growth, they also affect the formation of the soil structure, soil biodiversity, and soil physical stability of the resistance to erosion, and so on (Kay & Rainer, 2008). Soil nitrogen and phosphorus are important parts of the geobiochemical circulation. The unreasonable distribution of soil nitrogen and phosphorus is an important factor causing eutrophication in permanent wetlands such as rivers and lakes (Li et al., 2008). The enrichment nutrients in water body such as N and P, lead to some algae proliferate abnormally. As the results of transparency of water and dissolved oxygen decreasing, a large number of aquatic plants died. With the problems of nonpoint source pollution and eutrophication becoming increasingly serious, research on the spatial heterogeneity distribution features of STN and STP at the watershed scale has became a focus in the current environmental and agroforestry field (Dobos, Micheli, & Montanarella, 2007). In recent years, the employment of geographic information system (GIS) and geostatistics to study the spatial distribution characteristics and to reveal the spatial variation laws and its influencing factors has garnered more attention.

Researchers have conducted many studies about the spatial distribution characteristics of soil nutrients, and the results showed that the spatial distribution of STN and STP exhibited random or structured spatial variation characteristic because of the significantly different soil physical, chemical, and biological processes in different directions (Wang, 1999; Wang, Zhang, Yu, & Zhang, 2006). The research scope involved different scales. Initially, geostatistics was applied to study the spatial heterogeneity distribution and characteristics of soil nutrients by researches. The spatial correlation of soil nutrients in Hawaii was analyzed by geostatistics, and the results showed that the spatial distance of soil phosphorus, potassium, calcium, and magnesium content was between 32 and 42 km (Yost, Uehara, & Fox, 1982). In 1997, geostatistical method was used to study the soil spatial changes in the mid‐west Taiwan (Chien, Lee, Guo, & Houng, 1997). Soil zinc map of the USA was analyzed using geostatistics, GISs, which concluded that the correlation distance of total zinc was 480 km (White, Welch, & Norvell, 1997). One hundred and six soil samples were collected and analyzed for soil organic matter, STN, available N, available P and available K, and the spatial variation in these soil nutrients was quantitatively analyzed by geostatistical methods, and the contour maps of each nutrient were drawn (Zhang, 1998). The spatial pattern characteristics of soil carbon and nitrogen in the turkey lakes watershed were studied employing the regression analysis (Creed, Trick, Band, & Morrison, 2002). In 2005, many studies based on the geostatistics were conducted to study the soil spatial variations in the Senegal Valley and found clear spatial variations in soil nutrients (Haefelel & Wopereis, 2005). Gradually, the GIS was used to predict the site indices in the studies of the spatial distribution of different soil nutrients. The spatial heterogeneity distribution of soil nutrients was researched employing GIS and geostatistics, and the results indicated that different soil nutrients had obvious different spatial variation patterns and different spatial correlations (Cambardella et al., 1994). In 2006, the digital elevation model (DEM) was employed to evaluate the spatial heterogeneity distribution of soil carbon and nitrogen in a Japanese cedar forest, and the researchers found that using DEM greatly facilitated the research on soil carbon and nitrogen (Kotaro, 2006). The GIS based on geostatistical method was applied to analyze the spatial variance characteristics of STP in Chaohu lake watershed and the contribution factors. The results showed that the semivariogram model of STP followed exponential model and the STP had a moderate spatial autocorrelation (Zhou, Gao, Sun, Zhao, & Zhang, 2007). In the study of soil nutrients of forest in Badaling, the results showed that different vegetation types corresponded to different spatial distributions of the soil nutrients (Yu, Zhang, & Zhu, 2009). The spatial distribution of soil organic carbon (SOC) in the Yaoxiang small watershed diminished gradually from the eastern and northwestern parts to central and southern parts, which was basically consistent with the trend of DEM in the Yaoxiang small watershed (Zhang, Gao, Wang, Liu, & Li, 2015). In brief, scholars have focused on the spatial distribution features of soil nitrogen and phosphorus employing different methods and technologies. Many researches have mainly concentrated on the spatial distribution of SOC, STN, and STP under different regions and vegetation types, and the main research areas distributed along the Loess Plateau, the Yangtze River basin, and the southwest region of China. The black soil in northeastern China has also been studied, but the research results about the spatial heterogeneity and distribution rules of soil nitrogen and phosphorus have seldom been reported in the northern mountainous area of China.

The study area belongs to a typical mountainous area in northern China, with serious soil and water erosion. Since the 1980s, with the implementation of the Return Farmland to Forests Project, many afforestation vegetation types have been developed, and many researches have been written on ecological afforestation technologies and soil hydrological benefits (Wan, Ji, & Gu, 2014; Yang, Hu, & Luo, 2011). However, there are generally few reports on the soil improvement benefits of the Return Farmland to Forests Project, and it is not beneficial to assess the ecological effects of the Return Farmland to Forests Project. In this paper, we selected the Yaoxiang watershed in southern Shandong Province as a research site. Using GIS and geostatistics, our goal was to achieve the following objectives: (1) to analyze the influences of slope, slope direction, and elevation on STN and STP, and establish the regression equation between elevation and the content of STN and STP; (2) to discuss the influences of forest vegetation coverage, elevation, and other topographic factors on the spatial heterogeneity distribution; and (3) to reveal the spatial heterogeneity distribution of STN and STP at the watershed scale using Kriging interpolation. We hope this can provide the theoretical basis for the construction of the Return Farmland to Forests Project and the evaluation of the forest ecological service function in the mountainous lands of northern China.

## 2 Materials and Methods

### 2.1 Study area condition

**Figure 1:**
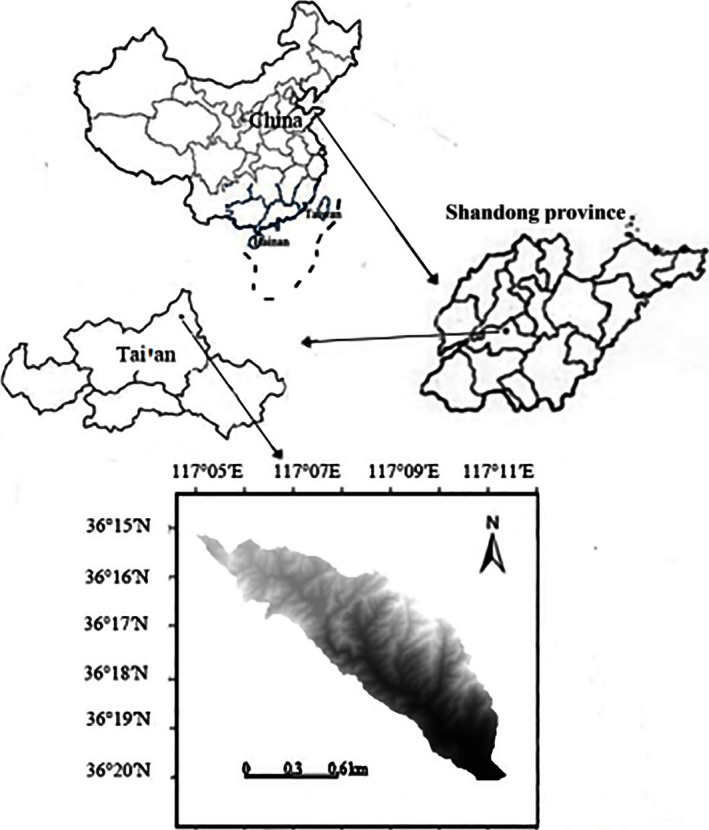
Geographic location map of the study area. Yaoxiang watershed is located in the north of Tai'an

The Yaoxiang watershed is located in Tai'an (36°15′58″–36°20′30″N and 117°05′39″–117°11′26″E) City of Shandong Province, with a total area of 4.20 km^2^ (Fig. 1) and elevations ranging from 310 to 950 m, which is belonging to a typical mountainous area in northern China. The topographic feature of the Yaoxiang watershed is high in the northwest and low in the southeast. The climate is semiarid with an average annual precipitation of 758 mm. The annual average air temperature is 14.5°C. The frostless season is 196 days. The soil type is referred to Brown soil; the thickness of the soil layer is 15–20 cm, and it is subject to high soil and water loss. According to the results of a floristic‐vegetational analysis (CAS, 2004), the vegetation types of the study area belong to coniferous forests and deciduous broad‐leaved forests in the warm temperate zone, including arbor species: *Pinus densiflora Sieb. et Zucc*,* Larix kaempferi*,* Quercus acutissima*,* Castanea mollissima*, and *Robinia pseudoacacia*; the main shrubbery species include *Lespedeza bicolor Turcz*,* Vitis amurensis*,* Spiraea trilobata*,* Spiraea fritschiana Schneid,* and *Ziziphus jujuba var. spinose* (Bunge) Hu; the herbaceous species include *Themeda triandra Forsk.var. Japonic*,* Zoysia japonica*,* Bothriochloa ischaemum* (L.) Keng, and *Artemisia lavandulaefolia* DC.

### 2.2 Sampling and processing

**Figure 2:**
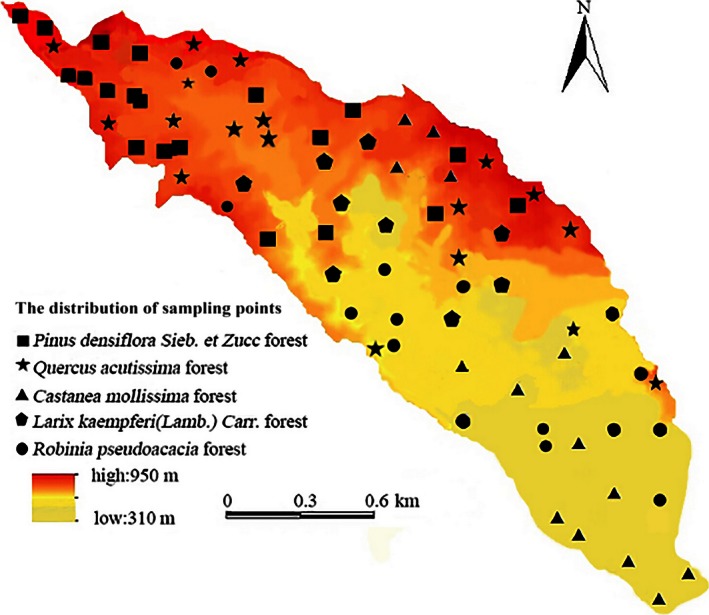
Map of soil sample points distribution in Yaoxiang watershed. Different colors represent different elevations, the deeper the color, the higher is the elevation

This work was based on the “Observation Methodology for Long‐term Forest Ecosystem Research,” which was the Forestry Standards of the People's Republic of China (LY/T 1952–2011) (Wang, Lu, Li, & Niu, 2011). The sampling plots were designed based on the topographic features and vegetation types (Table 1) of the Yaoxiang watershed, according to a DEM grid graph generated by a 1:1,000 topographic map (10 × 10 m) and combined with land‐use data interpreted from remote sensing images in the Yaoxiang watershed. In November 2014, a total of 77 sampling plots were selected in the study area (Fig. 2), and a total of 154 soil samples were collected from the depth of 0–10 and 10–30 cm. Meanwhile, GPS was used to record the longitude, latitude, and elevation values. After collecting the samples, they were taken to the laboratory by the processes of air drying, fine grinding, and sieving (2.000‐, 0.250‐, and 0.149‐mm soil sieve). STN was measured using the semimicro‐Kjeldahl determination (Li, Chen, & Zhao, 2006; Xie et al., 2012), and STP was measured using fused sodium hydroxide with the molybdenum stibium antireagent color method (Nelson & Sommers, 1982).

**Table 1:** Basic status of typical forest stands of the study area

Vegetation types	Tree age (year)	Tree hight (m)	DBH (cm)	Canopy density (%)	Elevation (m)	Soil texture
*Robinia pseudoacacia* forest	16–21	11.3	9.61	79	389–850	Brown soil
*Quercus acutissima* forest	15–23	13.1	11.27	81	450–890	Brown soil
*Pinus densiflora Sieb. et Zucc* forest	20–30	11.8	13.02	82	510–950	Brown soil
*Larix kaempferi(Lamb.) Carr*. forest	22–34	15.2	12.08	75	410–840	Brown soil
*Castanea mollissima* forest	15–20	4.9	9.4	77	310–560	Brown soil

DBH refers to the diameter at breast height of the tree.

### 2.3 Spatial heterogeneity analysis

Conventional statistical analysis: SPSS 19.0 software was used for classical statistical and correlation analysis.

Geostatistical analysis: ArcGIS 10.0 software was used to establish the Yaoxiang watershed DEM and extract the topographic factors. The extracted topographic indexes mainly included slope and slope direction. The semivariance analysis of STN and STP could be completed in GS + 9.0. On this basis, the ordinary kriging interpolation prediction analysis could be performed in ArcGIS 10.0 software. Finally, the spatial prediction distribution maps of STN and STP in the Yaoxiang watershed were produced.

The formula of the semivariance function was calculated with equation (1) (Jiang, Liang, & Li, 2005).

(1) $$\gamma \left(h\right)=\frac{1}{2N(h)}\sum_{i=1}^{N(h)}{\left(z\left(x_{i}+h\right)-z\left(x_{i}\right)\right)}^{2}$$

where *γ*(*h*) is the experimental semivariogram value at a distance interval *h*;* h* is the distance interval; *N*(*h*) is the number of sample value pairs within the distance interval *h*;* z*(*x*_*i*_) and *z*(*x*_*i*_ + *h*) are the sample values at two points (*x*_*i*_ and *x*_*i*_ + *h*) separated by the distance interval *h*.

The semivariance function has five theoretical models and four indicators to describe the spatial correlation of variables, including the exponential model, gaussian model, spherical model, pure nugget effect model, and linear model. *C*_0_ is the nugget, *C*_1_ is the partial sill, *C*_0_ + *C*_1_ is the sill, and *C*_0_/(*C*_0_ + *C*_1_) is the ratio of the nugget to the sill. Along with the range, they are the parameters that characterize the spatial structure of a soil property.

With the increase in the distance interval *h*, the semivariance increases from a nonzero value to a constant value. The value of *C*_0_/(*C*_0_ + *C*_1_) indicates the degree of variation in the soil. The higher the value of *C*_0_/(*C*_0_ + *C*_1_) is, the higher degree of variation that is caused by the random part; on the contrary, the variation is mainly caused by the structural part (Peter & Andreas, 2015; Stacey, Lark, Whitmore, & Milne, 2006). If the value of *C*_0_/(*C*_0_ + *C*_1_) is smaller than 25%, the variable has intense spatial correlation, between 25% and 75%, the variable shows medium spatial correlation; if the value is larger than 75%, the spatial correlation is weak (Li et al., 2008; Mackey et al., 2000; Zhao, Liu, Sui, Zhang, & Meng, 2006). The range indicates the spatial dependent distance; when the distance of the samples is close to it, the variable has spatial correlation; on the contrary, the spatial correlation does not exist between the samples if the distance exceeds the range (Liu, Li, Yang, & Xun, 2012; Moore, Gessler, Nielsen, & Peterson, 1993).

The soil fractal dimension (*D*): *D* is used to calculate the complexity of the variable factors (Li, Lei, & Wang, 2000). There is linear relationship between *D* and a double logarithmic relationship (log *γ*(*h*) ∝ log *h*) of the semivariance function within a distance interval *h*; the slope (*M*) of the regression line on the different spatial distance intervals can be obtained after the linear regression. *D* is calculated with equation (2).

(2) $$D=\frac{4-M}{2}$$

where *D* is the soil fractal dimension indicating the degree of structural heterogeneity between soil samples. The larger the value of *D* is, the smaller the degree of heterogeneity between the soil samples and the greater the degree of homogeneity. Otherwise, the degree of heterogeneity between samples is great and the homogeneity is low.

## 3 Results

### 3.1 Conventional statistical analysis of STN and STP in the Yaoxiang watershed

According to the statistical results of STN and STP in the Yaoxiang watershed (Table 2), Table 2 showed that the STN and STP contents in the 0‐ to 10‐cm soil layer were greater than those in the 10‐ to 30‐cm soil layer. The soil variation coefficient (*C*_v_) of STN and STP (42.25% and 14.77%, respectively) in the 0‐ to 10‐cm soil layer was greater than those in the 10‐ to 30‐cm soil layer (28.77% and 11.60%, respectively). The analysis of variance indicated that the STN content was significantly different between the 0‐ to 10‐cm and 10‐ to 30‐cm soil layers (*p* < .01), and the STP content was different between the 0‐ to 10‐cm and 10‐ to 30‐cm soil layers (*p* < .05).

**Table 2:** Conventional statistical analysis of soil total nitrogen (STN) and total phosphorus (STP) in Yaoxiang watershed

Soil layer(cm)	Soil nutrient	Mean (g/kg)	Minimum (g/kg)	Maximum (g/kg)	Standard deviation	*C*_v_ (%)
0–10	STN	3.29 (A/a)	1.46	6.18	1.39	42.25
	STP	0.88 (A/a)	0.78	1.04	0.13	14.77
10–30	STN	2.34 (B/b)	1.5	3.89	0.67	28.77
	STP	0.69 (A/b)	0.37	0.84	0.08	11.60

Coefficient of variation (*C*_v_) is the ratio of standard deviation to average, capital letters indicate the significant differences between the 0‐ to 10‐cm and 10‐ to 30‐cm soil layer (*p* < .01); lower case letters mean significance between the 0‐ to 10‐cm and 10‐ to 30‐cm soil layer (*p* < .05). The same below.

### 3.2 Semivariance analysis of STN and STP in the Yaoxiang watershed

**Figure 3:**
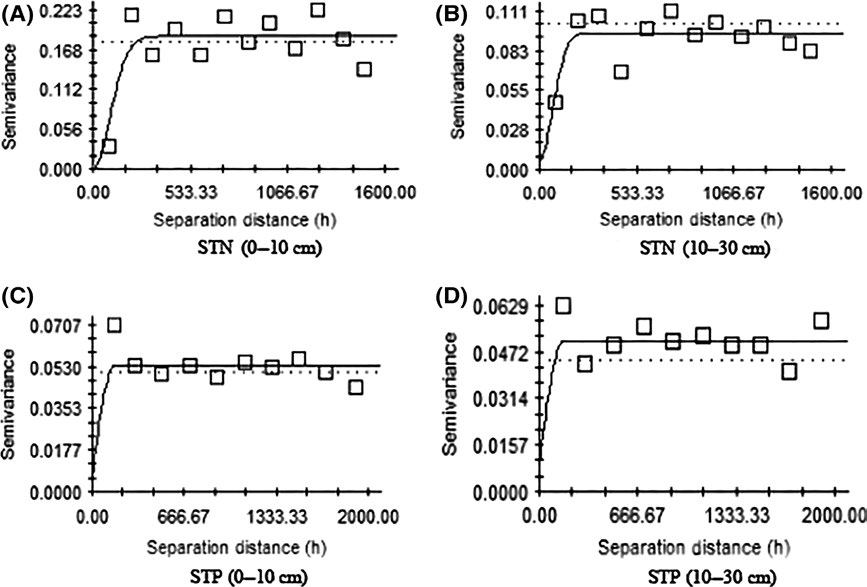
Semivariogram analysis of soil total nitrogen and total phosphorus contents in Yaoxiang watershed by ordinary kriging method

The choice of semivariance model is the key to analyze the spatial heterogeneity distribution of STN and STP. By comparing different parameters, the spherical model and exponential model were selected as the perfect models of STN and STP, respectively. From the results of the semivariance model analysis (Table 3, Fig. 3), it could be concluded that the maximum *C*_0_/(*C*_0_ + *C*_1_) of STN and STP in the 0‐ to 10‐cm and 10‐ to 30‐cm soil layers was 23.2%. Moreover, the values of *C*_0_/(*C*_0_ + *C*_1_) of STN and STP in the 0‐ to 10‐cm soil layer were all higher than those in the 10‐ to 30‐cm soil layer, and the value of *C*_0_/(*C*_0_ + *C*_1_) of STN was higher than that of STP. These results indicated that there was strong spatial distribution autocorrelation in STN and STP, and less spatial correlation existed in the surface soil (0–10 cm) than in the 10‐ to 30‐cm soil layer, and STP showed more intense spatial correlation than STN. So it was consistent with the conventional statistical analysis of STN and STP above (Table 2). In addition, the step length of the semivariance analysis in our study was 50 m, and the range (159–239 m) was much greater than the step length. Therefore, the results obtained from the ordinary kriging interpolation were relatively accurate. Moreover, the determination coefficients (*R* ^2^ = .874–.912) of the semivariance model analysis of STN and STP were large, which demonstrated that the fitting results of the semivariance theory model were very ideal.

**Table 3:** Semivariogram analysis of soil total nitrogen (STN) and total phosphorus (STP) in Yaoxiang watershed by ordinary kriging method

Soil nutrient	Soil layer (cm)	Theoretical model	*C*_0_	*C*_0_ + *C*_1_	*C*_0_·(*C*_0_ * + C*_1_)^−1^) (%)	Range (m)	*R* ^2^
STN	0–10	Spherical model	0.165	0.711	23.2	159	.897
	10–30	Spherical model	0.154	0.885	17.4	180	.912
STP	0–10	Index model	0.085	0.459	18.6	239	.874
	10–30	Index model	0.037	0.387	9.5	198	.892

*C*_0_ is the nugget, *C*_1_ is the partial sill, *C*_0_ +* C*_1_ is the sill, and *C*_0_/(*C*_0_ +* C*_1_) is the ratio of the nugget to the sill. *R* ^2^ is the determination coefficients.

### 3.3 Correlation analysis between topographic indexes and STN and STP contents in the Yaoxiang watershed

**Figure 4:**
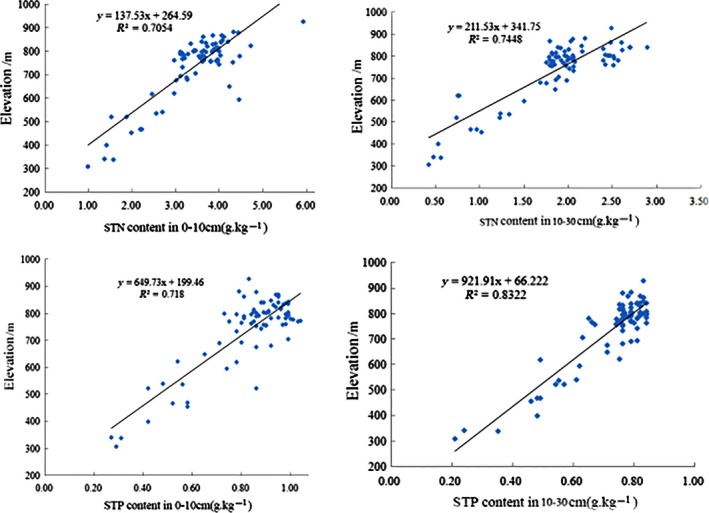
Correlation analysis of soil total nitrogen and total phosphorus with elevation in Yaoxiang watershed

From the Table 4 and Fig. 4, it could be concluded that STN and STP had significantly positive correlation with elevation (correlation coefficients were .495 and .425, respectively), and that the content of STN and STP increased with the increase in elevation in Yaoxiang watershed; the content of STN and STP had positive correlation with cosine slope (correlation coefficients were .329 and .259, respectively), and it showed that the content of STN and STP increased with the degree of slope direction to the north increasing; the content of STN and STP had negative correlation with sine slope (correlation coefficient was −.199 and −.215, respectively), and it showed that the content of STN and STP increased with the degree of slope direction to the west increasing (Jia, Duan, & Qiao, 2007). On the other hand, there was less correlation between STN and STP and slope. Therefore, we could conclude that the STN and STP are mainly distributed in the northwest of the Yaoxiang watershed.

**Table 4:** Correlations between topographic indexes and soil total nitrogen (STN) and total phosphorus (STP) contents

Soil nutrient	Elevation	Cosine slope	Sine slope	Slope
STN	.495**	.339**	−.199*	.115
STP	.425**	.259*	−.215*	.043

*Indicates that the columns are significantly different at *p* < .05; ** indicates that the columns are significantly different at *p* < .01.

Using multiple linear regression method, topographic indexes were used to explain and predict soil nutrient spatial variation. The regression equations are as follows:

(3) $$Y_{\mathrm{STN}}=2.238X_{1}+2.055X_{2}+1.968X_{3}+7.851,\;r^{2}=.688^{**}$$

(4) $$Y_{\mathrm{STP}}=1.183X_{1}+1.002X_{2}+0.577X_{3}+4.354,\;r^{2}=.518^{**}$$

where *Y*_STN_ is the STN content (g/kg) and *Y*_STP_ is the STP content (g/kg); *X*_1_ is the elevation, *X*_2_ is the sine slope, and *X*_3_ is the cosine slope.

From the regression equation, we could know that the topographic indexes, such as elevation, cosine slope, and sine slope, were all related with the spatial distribution of STN and STP (fitting decision coefficients are .688 and .518, respectively), the fitting effect of the equation was good.

### 3.4 Soil fractal dimension (*D*) of STN and STP in the Yaoxiang watershed

From the full‐range view, Table 5 showed that the value of *D* for STN and STP in the 0‐ to 10‐cm (1.879 and 1.929, respectively) soil layer was smaller than that in the 10‐ to 30‐cm soil layer (1.931 and 1.977, respectively), demonstrating that the surface soil (0–10 cm) had a greater degree of spatial heterogeneity. Moreover, the *D* values of STN were lower than those of STP, demonstrating that STN had a greater degree of spatial heterogeneity. These results were consistent with the results of the statistical analysis and semivariance analysis. From the view of different directions, the *D* values of STN and STP were low in the northwest–southeast direction (NW–SE) and high in the south–north direction (S–N). This indicated that the spatial heterogeneity distribution of STN and STP were significant in the NW–SE direction but not in the S–N direction; therefore, the spatial distribution pattern was not obvious in the S–N direction. This was relatively consistent with the slope direction in the Yaoxiang watershed.

**Table 5:** Soil fractal dimension (*D*) of soil total nitrogen (STN) and total phosphorus (STP) contents in each soil layer in Yaoxiang watershed

Orientation	STN		STP	
	0–10 cm	10–30 cm	0–10 cm	10–30 cm
	*D*	*D*	*D*	*D*
S–N	1.914	1.975	1.948	1.985
NE–SW	1.854	1.932	1.941	1.972
E–W	1.859	1.897	1.891	1.914
NW–SE	1.796	1.847	1.841	1.854
Full‐range	1.879	1.931	1.929	1.977

The values of *D* indicate the spatial heterogeneity distribution degree in the different section. S–N, south–north section; NE–SW, northeast–southwest section; E–W, east–west section; NW–SE, northwest–southeast section.

### 3.5 Spatial distribution of STN and STP in the Yaoxiang watershed

**Figure 5:**
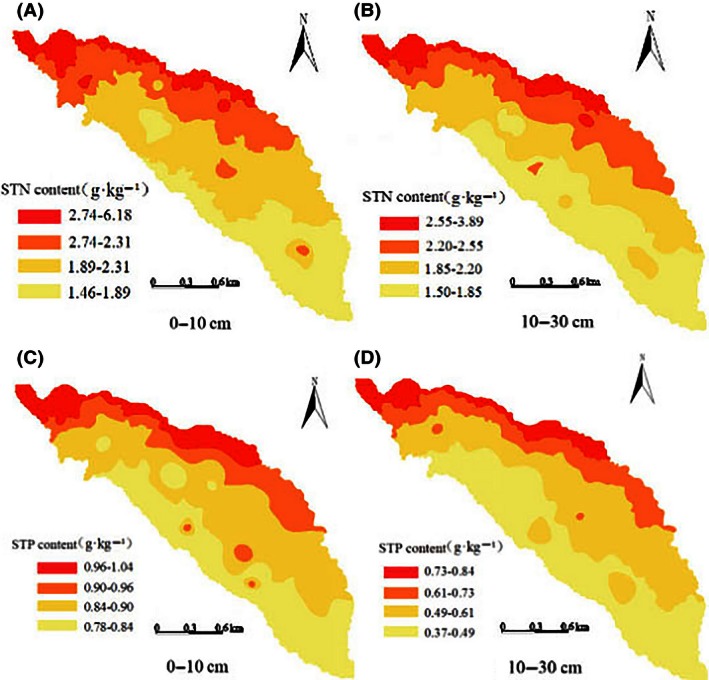
Distribution map of soil total nitrogen (A, B) and soil total phosphorus contents (C, D) of different soil layers in Yaoxiang watershed by ordinary kriging method

Figure 5 showed that the overall distribution of the STN and STP contents in the Yaoxiang watershed decreased from the northwest to the southeast, and the distribution followed the same trend with DEM in the Yaoxiang watershed. Moreover, STN had high correlation with STP, and the correlation coefficient (*r* ^2^) was .711. The regression analysis result reflected a significant linear relationship between STN and STP, and the regression equation is:

(5) $$Y_{\mathrm{STN}}=3.569\times X_{\mathrm{STP}}-0.140,\;r^{2}=.711^{**}$$

where *Y*_STN_ is the STN content (g/kg) and *X*_STP_ is the STP content (g/kg).

## 4 Discussion

### 4.1 Variation characteristics of STN and STP in different soil layers in the Yaoxiang watershed

There have been many studies on the distribution of soil nutrients in different soil depths (Hengl, Heuvelink, & Stein, 2004; Kay & Rainer, 2008; Kong, Zhang, Kou, Liu, & He, 2014). Studies about the SOC, STN, and STP distribution of typical vegetation riparian zones in the upper reaches of the Hun River have been conducted by Kong et al. (2014), and they showed that the SOC, STN, and STP contents decreased with the increase in soil layer depth. For the spatial variation in soil, the spatial heterogeneity distribution of SOC and other nutrients in the forestlands of the mountainous area of Changbai Mountain was studied, and the results showed that the SOC content varied with the soil depth and that the spatial variability of soil on the surface was greater than that in deep soil (Liu, 2013). In our study, we found that the STN and STP contents in the Yaoxiang watershed varied with the soil layer, and the STN and STP contents in the 0‐ to 10‐cm soil layer were greater than those in the 10‐ to 30‐cm soil layer. This result can be potentially explained by the large amount of forest litter in the Yaoxiang watershed. Litter is an important material base of the circulation of matter and the main supply of nitrogen and phosphorus in the ecosystem. The 0‐ to 10‐cm soil layer covered more litter that falling from the forest vegetation, which were decomposed in the soil and releasing more nitrogen and phosphorus nutrients. They had beneficial effects on the soil and increased the source of nutrients in the soil, which leads to the conclusion that STN and STP contents in the 0‐ to 10‐cm soil layer were greater than those in the 10‐ to 30‐cm and had a significant effect. Moreover, the *C*_v_ of STN was greater than that of STP. In general, when *C*_v_ < 10%, there is weak variability; when *C*_v_ is between 10% and 100%, there is moderate variability; and when *C*_v_ > 100%, there is strong variability (Li et al., 2008). The maximum *C*_0_/(*C*_0_ +* C*_1_) of STN and STP in the soil layers was 23.2%; therefore, STN and STP had moderate variability in the Yaoxiang watershed. Our study also found that the *C*_v_ of STN and STP in the 0‐ to 10‐cm soil layer was greater than that in the 10‐ to 30‐cm soil layer. This could be potentially explained by the effects of environmental factors such as topographic and human factors, which greatly damaged the surface soil structure (0–10 cm). However, the 10‐ to 30‐cm soil layer experienced less interference by artificial factors; therefore, the value of *C*_v_ of soil in the 10‐ to 30‐cm soil layer was smaller.

### 4.2 Spatial distribution analysis and the factors influencing STN and STP in the Yaoxiang watershed

Soil nutrients, such as nitrogen and phosphorus, are comprehensively affected by natural and human factors such as topography, vegetation, climate, land use and management measures. Various factors jointly determine the spatial distribution pattern and the formation of the soil, and they also determine the transformation direction and rate of change of soil nutrients (Liu et al., 2015). In the research on the nutrient characteristics of forest soil at different elevations in the Fanjingshan Nature Reserve, researchers found that the nutrients showed clear vertical distribution characteristics with the increase in altitude (Yan, Zhang, Shi, Lin, & He, 2015). Through the research about the spatial variability of soil nitrogen and the affecting factors in the hilly area of the Mid‐Sichuan Basin, it was found that the spatial distribution of STN showed similar trend with STP; both presented a low trend from north to south as the topography changed (Luo et al., 2015). In our research, the spatial distribution tendency of STN and STP decreased from the northwest to the southeast, and it was consistent with the DEM map (Fig. 2) in the Yaoxiang watershed. Therefore, the spatial distribution of STN and STP had close correlation with the elevation in the Yaoxiang watershed.

Moreover, because the maximum *C*_0_/(*C*_0_ +* C*_1_) of STN and STP in the soil layers is less than 25%, this indicates that a strong spatial distribution autocorrelation existed in STN and STP in the Yaoxiang watershed. Structural factors such as topography and vegetation were the main factors influencing the spatial heterogeneity distribution of STN and STP, whereas the influence of random factors was smaller. The values of *C*_0_/(*C*_0_ +* C*_1_) of STN and STP in the 0‐ to 10‐cm soil layer were all higher than those in the 10‐ to 30‐cm soil layer, indicating that there was less spatial correlation in the surface soil (0–10 cm) than in the 10‐ to 30‐cm soil layer. From Table 3, it can also be observed that the value of *C*_0_/(*C*_0_ +* C*_1_) of STN was higher than that of STP. Therefore, STP showed more intense spatial correlation than STN. The spatial heterogeneity distribution of STP was mainly affected by structural factors, and its change was relatively stable (Peter & Andreas, 2015; Stacey et al., 2006).

At the same time, the spatial heterogeneity distribution of STN and STP had a close relationship with the vegetation in the Yaoxiang watershed. First, in the northwest part (which has a high elevation) of the watershed, after years of afforestation, the forest vegetation has been less destroyed by humans. Therefore, the tree litter, acting as an effective source of soil nutrients, improved the soil structure and physical and chemical properties, resulting in high STN and STP contents in the northwest part. Second, the northwest part of the watershed was covered with *Pinus densiflora Sieb. et Zucc*. or the mixed forests of *Pinus densiflora Sieb. et Zucc* and *Q. acutissima* (Fig. 1; Table 1). Therefore, northwest had high vegetation coverage, suitable temperature, and developed humidity plant roots. Litter decomposition in the northwest was beneficial and conducive for the accumulation of soil nitrogen and phosphorus (Liang, Yuan, & Lin, 2006; Liu, Fu, & Wu, 2003). Meanwhile, the southeast part of the watershed was mainly covered with *C. mollissima* and *R. pseudoacacia* and was disturbed by human activities in this area, resulting in much lower STN and STP contents than in the northwest part. Thus, we should develop comprehensive measures to reduce human interference and destruction to the forest vegetation and strengthen its preservation and restoration; it is very important for the storage and accumulation of nitrogen and phosphorus in the mountain watershed.

## 5 Conclusions

1. The STN and STP contents in different soil layers of the Yaoxiang watershed were significantly different, and they were all of medium variation. In particular, the variation in STN was greater than that of STP.
2. The STN and STP contents of the Yaoxiang watershed showed intense spatial autocorrelation. Structural factors, such as the elevation, slope, slope direction, and vegetation type, were the main factors that influencing the content and distribution of STN and STP of the Yaoxiang watershed.
3. The distribution trend of STN and STP in the Yaoxiang watershed was higher in the northwest part than that in the southeast part, and it decreased from the northwest to the southeast. This result was similar to the trend in the watershed DEM.
4. The results demonstrated that the ordinary kriging method was suitable for studying SOC and STN spatial distribution in the Return Farmland to Forests Project in the mountainous lands of northern China and the other similar parts of the world.

## Data Accessibility

Measurements of all contents of STN and STP, values of DEM‐derived variables at sampling locations used to extract its topographic factors could access these data in the database of Shandong Provincial Key Laboratory of Soil Erosion and Ecological Restoration.

## Conflict of Interest

The authors have no conflict of interests to declare.

## Funding Information

The major water conservancy scientific research and technology promotion projects of Shandong province (2014–2016), the Special Fund for Forestry Scientific Research in the Public Interest (Grant/Award Number: Nos. 201204101, 201404303), Beijing Municipal Education Commission (Grant/Award Number: CEFF‐PXM2016‐014207‐000038).
